# Supplementary material for: Splice-Junction-Based Mapping of Alternative Isoforms in the Human Proteome
Source: Cell Rep. Author manuscript; Available in PMC 2020 Jan 15. (PMC6961840; doi:10.1016/j.celrep.2019.11.026)
Supplement: 2 [file NIHMS1546469-supplement-2.zip › DF1/PXD010154/AdrenalGland/AdrenalGland_2_EPB41L3_VIFLQQGSAPFLESQK.pdf]

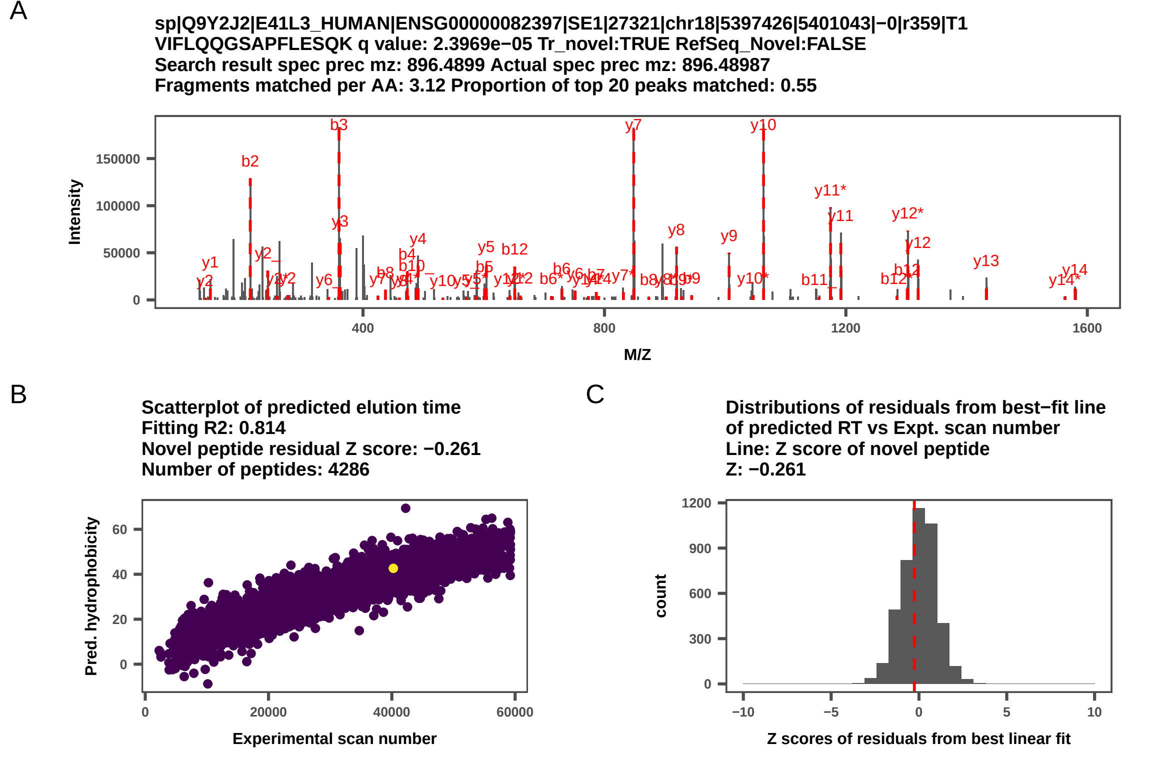

A
sp|Q9Y2J2|E41L3_HUMAN|ENSG00000082397|SE1|27321|chr18|5397426|5401043|−0|r359|T1
VIFLQQGSAPFLESQK q value: 2.3969e−05 Tr_novel:TRUE RefSeq_Novel:FALSE
Search result spec prec mz: 896.4899 Actual spec prec mz: 896.48987
Fragments matched per AA: 3.12 Proportion of top 20 peaks matched: 0.55
Intensity
M/Z
b2
b3
y3
y7
y10
y11*
y11
y12*
y12
y8
y9
y13
y14
y4
y1
0
50000
100000
150000
400
800
1200
1600
B
Scatterplot of predicted elution time
Fitting R2: 0.814
Novel peptide residual Z score: −0.261
Number of peptides: 4286
Pred. hydrophobicity
Experimental scan number
0
20
40
60
20000
40000
60000
C
Distributions of residuals from best−fit line
of predicted RT vs Expt. scan number
Line: Z score of novel peptide
Z: −0.261
count
Z scores of residuals from best linear fit
300
600
900
1200
−10
−5
5
10
